# Detailed Analysis of the Palmomental Reflex and Its Clinical Significance

**DOI:** 10.1002/brb3.70164

**Published:** 2024-11-22

**Authors:** Benxu Ma, Jianying Zhang, Yanlei Cui, Huanmin Gao

**Affiliations:** ^1^ Department of Acupuncture Qingdao Central Hospital, University of Health and Rehabilitation Sciences (Qingdao Central Hospital) Qingdao China; ^2^ Department of Neurology The Affiliated Hospital of Qingdao Binhai University Qingdao China

**Keywords:** clinical significance, neurological disorders, palmomental reflex (PMR)

## Abstract

**Purpose:**

This comprehensive review thoroughly explores the clinical significance of the palmomental reflex (PMR) in neurological disorders. PMR is a primitive reflex that, when reemerging in adults, indicates underlying neurological dysfunction.

**Method:**

The article elaborates on the clinical assessment techniques, neurophysiological basis, and applications of PMR in various neurological disorders, including neurodegenerative diseases, cerebrovascular disorders, traumatic brain injury, and multiple sclerosis.

**Finding:**

By understanding the modulation and suppression mechanisms of PMR, valuable insights into the specific neurological impairments associated with these disorders can be gained.

**Conclusion:**

The potential of PMR as a diagnostic marker, prognostic indicator, and treatment monitoring tool is increasingly evident.

## Graphical abstract

The palmomental reflex, a primitive reflex typically absent in adults, reemerges in various neurological disorders. Its presence and characteristics offer valuable insights for diagnosis, disease progression assessment, and treatment monitoring across conditions such as Parkinson's disease, Alzheimer's disease, stroke, and traumatic brain injury.

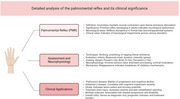

## 1 Introduction

The human nervous system is characterized by the presence of various primitive reflexes, which are typically observed in infancy but tend to diminish or disappear as the brain matures and inhibitory mechanisms develop (Touwen 1995). However, in certain neurological conditions, these primitive reflexes may reemerge, providing valuable clinical insights into underlying neurological dysfunction (Paulson and Gottlieb 1968). Among these reflexes, the palmomental reflex (PMR) has garnered significant attention due to its potential diagnostic and prognostic implications across a wide range of neurological disorders (Choi et al. 2011).

PMR, also known as the Marinescu–Radovici reflex or the Mendel–Bechterew reflex, refers to the involuntary contraction of the mentalis muscle (the muscle protruding the chin) in response to a firm, sustained stroke or scratch on the thenar eminence (the fleshy base of the thumb; Owen and Mulley 2002). This reflex is considered a pathological sign in adulthood, as it typically disappears after the first few years of life, when cortical inhibitory mechanisms become well‐established (Zafeiriou 2004).

The reemergence of PMR in adults is often associated with disruptions or dysfunctions in the neural pathways responsible for the inhibition and modulation of this primitive reflex (Reis 1961). Consequently, the presence of PMR may serve as an indicator of underlying neurological impairments, making it a potentially valuable diagnostic tool in various neurological conditions (Marterer‐Travniczek et al. 1992).

### 1.1 Historical Background

PMR was first described independently by several researchers in the late 19th and early 20th centuries, including Marinescu, Radovici, Mendel, and Bechterew (Dalby 1970). These pioneers observed the reflex response in infants and noted its subsequent disappearance during normal development (Gabelle et al. 2016). However, they also recognized the reappearance of PMR in certain neurological disorders, prompting further investigation into its clinical significance (Little and Masotti 1974).

Since its initial discovery, PMR has been extensively studied and documented in various neurological conditions, ranging from neurodegenerative diseases to stroke, traumatic brain injury (TBI), and multiple sclerosis (MS) (Vreeling 1994). Numerous research efforts have been dedicated to understanding the neuroanatomical and neurophysiological underpinnings of PMR, as well as its potential utility as a diagnostic and prognostic marker (Van Gijn 1977).

### 1.2 Clinical Relevance and Significance

The clinical relevance of PMR lies in its potential to provide insights into the functional integrity of specific neural pathways and brain regions involved in the inhibition and modulation of primitive reflexes (Brodal 1981). The presence of PMR in adulthood may indicate disruptions or dysfunction in frontal lobe regions, corticobulbar tracts, extrapyramidal systems, or cortical inhibitory mechanisms, all of which play crucial roles in the suppression of this reflex (Walterfang and Velakoulis 2005).

By assessing the presence and characteristics of PMR, clinicians can potentially gain valuable information about the underlying neurological impairments and the extent of neurological involvement in various disorders (Gossman and Jacobs 1980). Additionally, PMR may serve as a prognostic indicator, providing insights into disease progression, treatment response, and overall clinical outcomes in certain neurological conditions (Polunina 2011).

This comprehensive review aims to provide a comprehensive overview of PMR, including its clinical assessment, neurophysiological basis, and implications in various neurological disorders. By synthesizing the latest research findings and clinical perspectives, this review seeks to enhance the understanding and utility of PMR as a valuable diagnostic and prognostic tool in the field of neurology (Okuda et al. 2008).

## 2 Clinical Assessment of the Palmomental Reflex

The accurate and consistent assessment of PMR is crucial for its effective utilization as a diagnostic and prognostic marker in neurological disorders. This section outlines standardized techniques for eliciting and evaluating PMR, addressing potential confounding factors, and strategies for enhancing the accuracy of the assessment.

### 2.1 Elicitation Techniques

PMR is typically elicited by delivering a firm, sustained stroke or scratch to the thenar eminence (the fleshy base of the thumb) using a blunt object, such as the handle of a reflex hammer or a key (Lupescu et al. 2020). The stimulation should be applied with sufficient pressure to evoke a response while avoiding excessive discomfort or pain for the patient (Vidovic et al. 2021). Researchers have proposed various specific techniques for eliciting PMR, each with its unique advantages and considerations. Some of the commonly employed methods include:

1. Stroking technique: The examiner firmly strokes the thenar eminence in a unidirectional manner, applying consistent pressure and maintaining a steady pace (Wallace et al. 2021). The stroke should be sustained for several seconds, typically ranging from 5 to 10 s, to allow sufficient time for the reflex response to manifest (Melillo et al. 2023).
2. Scratching technique: An alternative approach involves scratching the thenar eminence with a blunt object, such as a key or the end of a reflex hammer (Abraham, Reinhart, and Svoboda 2002). The scratching motion should be firm and sustained, covering a small area of the thenar eminence for several seconds (Lenggenhager 2009).
3. Repetitive tapping technique: This technique involves delivering a series of firm taps or percussions with a blunt object to the thenar eminence, maintaining a consistent rhythm and pressure (Zanoni et al. 2023; Whittle and Miller 1987).

While these techniques have been widely employed, there is currently no consensus on the optimal method for eliciting PMR. The choice of technique may depend on factors such as examiner preference, patient comfort, and the specific clinical context (Oli and Shrestha 2024). Regardless of the technique used, it is essential to ensure consistency and standardization within and across clinical settings to facilitate accurate comparisons and interpretation of the reflex response.

### 2.2 Assessing the Reflex Response

The evaluation of PMR involves carefully observing and grading the contraction of the mentalis muscle, which is responsible for protruding the chin (Niedermeyer 1998). A positive PMR is characterized by a visible contraction or twitching of the mentalis muscle in response to the stimulation of the thenar eminence (Walther et al. 2013).

It is important to note that the reflex response may vary in intensity and duration, ranging from a brief, subtle contraction to a sustained, forceful contraction of the mentalis muscle (Gieysztor, Choińska, and Paprocka‐Borowicz 2018). The assessment of PMR should take into account both the presence and the characteristics of the reflex response, as these factors may provide valuable insights into the underlying neurological condition (Tankisi et al. 2021).

Several key aspects should be considered when assessing PMR. The examiner should note the time elapsed between the stimulation and the onset of the reflex response, as well as the duration of the contraction (Modrell and Tadi 2023). A prompt onset and prolonged duration of the reflex may suggest more severe neurological involvement (Pecuch et al. 2021). The intensity and strength of the mentalis muscle contraction should be evaluated, ranging from mild to pronounced or forceful contractions (Goyal et al. 2022). More intense contractions may be associated with greater neurological dysfunction or disinhibition of the reflex (Gramespacher et al. 2020). In some cases, the reflex response may extend beyond the mentalis muscle, involving other perioral or facial muscles, such as the orbicularis oris (muscles around the mouth) or the platysma (neck muscle) (Byers and Dodge 1967). The spread or radiation of the reflex to adjacent muscles should be documented, as it may provide additional insights into the extent of neurological involvement (Yadav, Mahale, and Pal 2018). The examiner should assess whether the reflex response is unilateral (occurring on one side of the face) or bilateral (occurring on both sides) (André 2023). Asymmetrical or unilateral responses may suggest localized or lateralized neurological dysfunction (Camarda et al. 2019). For example, a stronger PMR on the right side of the chin in response to stimulation of the right hand may indicate dysfunction in the left hemisphere of the brain.

To standardize the assessment and facilitate consistent comparisons, various grading systems have been proposed for evaluating the intensity and characteristics of PMR (Cattaneo and Pavesi 2014). One widely adopted grading system categorizes PMR into four levels based on the duration and strength of the reflex response (Tsuyusaki et al. 2016): Absent (no visible contraction of the mentalis muscle upon stimulation); Present (mild contraction of the mentalis muscle, lasting less than 5 s); Brisk (moderate‐to‐strong contraction of the mentalis muscle, lasting 5–10 s); and Persistent (sustained contraction of the mentalis muscle, lasting more than 10 s). By adhering to standardized grading systems and documenting the specific characteristics of the reflex response, clinicians can enhance the objectivity and reproducibility of PMR assessment, enabling more accurate comparisons and monitoring over time (de Noordhout and Delwaide 1988).

### 2.3 Addressing False Negatives and Confounding Factors

While the presence of PMR is generally considered a pathological sign in adulthood, the absence of the reflex does not necessarily indicate a lack of neurological impairment (Borg, Warwick, and Ahmed 2021). Various factors can potentially contribute to false‐negative results, where PMR is not elicited despite underlying neurological dysfunction (Dickson 1998). Recognizing and addressing these confounding factors is crucial for enhancing the accuracy and reliability of PMR assessment.

#### 2.3.1 Cognitive Impairment

Patients with significant cognitive impairment or decreased levels of consciousness may not exhibit PMR, even in the presence of neurological dysfunction (Ghosh Md et al. 2022). In such cases, the ability to follow instructions and maintain attention during the examination can be compromised, potentially leading to false‐negative results (Camarda et al. 2020).

#### 2.3.2 Muscular Weakness or Paresis

Neuromuscular disorders or other conditions resulting in significant muscular weakness or paresis may hinder the visible manifestation of PMR (Peddireddy et al. 2006). If the mentalis muscle or associated facial muscles are severely weakened, the reflex contraction may not be apparent, despite the underlying neurological impairment (Noda et al. 2015).

#### 2.3.3 Peripheral Neuropathy

In cases of severe peripheral neuropathy or damage to the sensory nerves supplying the thenar eminence, the afferent pathway necessary for eliciting PMR may be disrupted, leading to an absent reflex response (Klein et al. 1997).

#### 2.3.4 Examiner Experience and Technique

The experience and technique of the examiner play a crucial role in the accurate assessment of PMR (Burrell et al. 2016). Inexperienced examiners or those who do not employ proper stimulation techniques may fail to elicit the reflex, resulting in false‐negative findings (Clark et al. 1998). If the initial assessment yields a negative result, it is recommended to repeat the examination multiple times, varying the stimulation technique or applying stronger stimuli (Matuszkiewicz and Gałkowski 2021); to encourage the patient to remain attentive and engaged during the examination (Swapna et al. 2020); to evaluate the strength and function of the facial muscles, particularly the mentalis muscle; and to rule out muscular weakness as a contributing factor (De Renzi, Pieczuro, and Vignolo 1966). In cases of suspected peripheral neuropathy or sensory deficits, additional tests, such as electrophysiological studies or sensory examinations, may be warranted to assess the integrity of the afferent pathway (Lusins and Bender 1973). Moreover, examiners should be ensured to receive proper training and adhere to standardized protocols for eliciting and assessing PMR, minimizing variability and enhancing consistency (Khan et al. 2021). By recognizing and addressing these confounding factors and implementing appropriate strategies, clinicians can enhance the diagnostic accuracy and reliability of PMR assessment, ultimately contributing to more informed clinical decision‐making (Isakov et al. 1984).

## 3 Neurophysiological Basis of the Palmomental Reflex

Understanding the neurophysiological mechanisms underlying PMR is crucial for interpreting its clinical significance and implications in various neurological disorders. This section explores the neural pathways, brain regions, and neurotransmitter systems involved in the modulation and suppression of PMR, shedding light on the potential neurological underpinnings of its reemergence in adulthood.

### 3.1 Neural Pathways and Brain Regions

PMR is a complex reflex involving multiple neural pathways and brain regions responsible for its elicitation, modulation, and suppression (Dutta et al. 2016). The afferent pathway for PMR originates from the sensory neurons innervating the thenar eminence, specifically the palmar branch of the median nerve (Rosenbohm et al. 2014). These sensory fibers convey tactile information from the stimulated area to the spinal cord and subsequently to higher brain regions for processing and integration (Thompson and Thompson 2023). At the level of the spinal cord and brainstem, the afferent signals from the thenar eminence are relayed through various interneuronal connections and ascending pathways (Swash et al. 2020). The reticular formation, a network of nuclei within the brainstem, is thought to play a crucial role in modulating and integrating PMR response (Iwasaki 2006).

The frontal lobe, particularly the prefrontal cortex and supplementary motor areas, is believed to be a key region involved in the inhibition and modulation of PMR (Spreen and Risser 2003). These areas exert their influence on the reflex through descending corticobulbar pathways, which connect the frontal lobe to the brainstem and spinal cord (Alves et al. 2009). The basal ganglia, a group of subcortical nuclei, and the associated extrapyramidal system are also implicated in the modulation of PMR (Nicholson and Pereira 2002). Structures such as the caudate nucleus, putamen, and globus pallidus are thought to play a role in the inhibition and regulation of primitive reflexes, including PMR (Bala et al. 2023). However, the exact neural circuitry is not fully elucidated yet.

Various neurotransmitter systems are involved in the modulation and suppression of PMR, including dopaminergic, cholinergic, and GABAergic pathways (Vreeling et al. 1994). Disruptions or imbalances in these neurotransmitter systems may contribute to the disinhibition and reemergence of PMR in certain neurological conditions (Caccia et al. 1996).

While the precise neuroanatomical and neurochemical mechanisms underlying PMR are still under investigation, it is clear that this reflex involves a complex interplay of neural pathways, brain regions, and neurotransmitter systems (Barabas and Matthews 1997). The reemergence of PMR in adulthood may reflect disruptions or dysfunctions in any of these components, providing insights into the underlying neurological impairments (Shahed and Jankovic 2007).

### 3.2 Developmental Aspects and Inhibitory Mechanisms

PMR is one of several primitive reflexes that can be used to diagnose neurological disorders. Other primitive reflexes, such as the snout reflex and jaw reflex, may also be present in certain neurological conditions. However, PMR has been shown to be a particularly useful diagnostic tool in certain contexts, such as in the diagnosis of Parkinson's disease (PD).

The disappearance of PMR during typical childhood development is attributed to the maturation of cortical inhibitory mechanisms and the establishment of higher order control over primitive reflexes (Shargorodsky, Lin, and Gopen 2010). As the brain develops, the prefrontal cortex and associated neural networks responsible for inhibitory control become more efficient, effectively suppressing the expression of primitive reflexes like PMR (Prajjwal et al. 2023).Primitive reflexes such as the snout, jaw, and grasp reflexes may also provide insights into neurological dysfunction.

The reemergence of PMR in adulthood is often considered a manifestation of disinhibition or a release of these primitive reflexes from cortical control (Moura et al. 2023). This disinhibition may result from various neurological insults or degenerative processes that disrupt the inhibitory mechanisms or the neural pathways involved in the modulation of PMR (Camarda et al. 2018).

By understanding the developmental aspects and inhibitory mechanisms underlying PMR, researchers and clinicians can gain insights into the neurodevelopmental and neurodegenerative processes that may contribute to the reemergence of this reflex in various neurological disorders (Güney et al. 2023).

## 4 Palmomental Reflex in Neurological Disorders

PMR has been extensively studied and documented in a wide range of neurological disorders, providing valuable diagnostic and prognostic information. This section explores the clinical implications and significance of PMR in various neurological conditions, highlighting its potential utility as a marker of disease progression and treatment response. The characteristic features of PMR may vary across different neurological disorders. For example, the intensity and duration of PMR may be more pronounced in patients with PD compared to those with Alzheimer's disease (AD) (Aguilar Agudo, Herruzo Cabrera, and Pino Osuna 2021).

### 4.1 Parkinson's Disease

In PD, the presence of PMR has been consistently observed and is considered a common clinical finding (Mandelbaum and Marks 2020). The reemergence of PMR in PD is thought to be related to the degeneration of dopaminergic neurons in the substantia nigra and the subsequent disruption of frontal–subcortical circuits involved in the inhibition of primitive reflexes (Sanchez‐Ramos, Ortoll, and Paulson 1996).

The presence and severity of PMR in PD patients have been associated with disease progression, cognitive impairment, and the development of levodopa‐induced dyskinesias (Wibawa et al. 2023). Additionally, PMR has been proposed as a potential marker for the early detection of PD, as it may precede the onset of cardinal motor symptoms in some cases (Cruccu et al. 2005).

Recent studies have also explored the relationship between PMR and specific PD subtypes or phenotypes, suggesting that the presence and characteristics of the reflex may vary depending on the underlying neuropathological patterns (Sigafoos et al. 2021). By monitoring the evolution of PMR, clinicians gain valuable insights into disease progression and treatment response, enabling more informed clinical decision‐making.

### 4.2 Alzheimer's Disease and Other Dementias

PMR has been observed in patients with AD and other forms of dementia, potentially reflecting the cognitive and neurological changes associated with these neurodegenerative conditions (Burns, Jacoby, and Levy 1991). In AD, the presence of PMR is thought to be related to the degeneration of frontal lobe structures and the disruption of cortical inhibitory mechanisms responsible for suppressing primitive reflexes (Sabayan et al. 2012).

Studies have suggested that PMR may be more prevalent in the later stages of AD, when cognitive impairment and frontal lobe dysfunction are more pronounced (Karpenko and Keegan 2021). Additionally, the presence and severity of PMR have been correlated with the degree of cognitive impairment and functional decline in AD patients (Chiang et al. 2005).

In vascular dementia and other forms of dementia, PMR may arise from the disruption of frontal–subcortical circuits or the presence of subcortical lesions affecting the pathways involved in reflex modulation (Coebergh and Stanton 2020). The assessment of PMR can provide valuable insights into the underlying neuropathological processes and the extent of neurological involvement in these dementia subtypes.

### 4.3 Multiple System Atrophy

PMR has also been documented in other neurodegenerative disorders, such as multiple system atrophy (MSA) (Thomas 1994). In MSA, PMR has been associated with the degeneration of extrapyramidal and cerebellar systems (Novak and Tabrizi 2011; Melillo et al. 2022). By assessing the presence and characteristics of PMR in these neurodegenerative disorders, clinicians can gain insights into the underlying neuropathological processes and potentially differentiate between various subtypes or phenotypes (Frank, Pari, and Rossiter 2006).

### 4.4 Stroke

PMR is frequently observed in patients who have suffered from stroke, particularly in cases involving lesions or dysfunction in the frontal lobe, basal ganglia, or corticobulbar pathways (Schott and Rossor 2016). The presence of PMR in stroke patients has been associated with the location and extent of the lesion, as well as the severity of neurological impairment (Morningstar et al. 2005).

In ischemic stroke, PMR may be observed in the acute phase or may develop over time as a result of reorganization and neuroplasticity processes (Zhang et al. 2021). The appearance of PMR in these cases may reflect the disruption of inhibitory mechanisms or the disinhibition of primitive reflexes due to the lesion or dysfunction in specific brain regions (Damasceno et al. 2005).

In hemorrhagic stroke, the presence of PMR has been linked to the location and extent of the hematoma, particularly when involving subcortical or frontal lobe regions (Rao, Jackson, and Howard 1999). The assessment of PMR can provide valuable prognostic information and insights into the functional recovery and rehabilitation potential in stroke patients (Dünser et al. 2018).

### 4.5 Vascular Dementia and Cognitive Impairment

In vascular dementia and other forms of vascular cognitive impairment, PMR has been observed and may be related to the presence of subcortical lesions, white matter changes, or disruptions in frontal–subcortical circuits (Coebergh and Stanton 2020). The assessment of PMR can aid in the differential diagnosis of vascular cognitive impairment and may provide insights into the underlying neuropathological processes and the extent of neurological involvement.

### 4.6 Traumatic Brain Injury

PMR is commonly observed in patients who have suffered from TBI, particularly in cases involving frontal lobe damage or diffuse axonal injury (Martello 2023). The presence of PMR in TBI patients has been associated with the severity of the injury, cognitive impairment, and the development of chronic neurological deficits (Miles et al. 2021).

In mild TBI or concussion, PMR may be a transient finding, potentially reflecting a temporary disruption of inhibitory mechanisms or a functional diaschisis (Wijdicks 2021). However, in cases of moderate‐to‐severe TBI, the persistent presence of PMR may indicate more extensive structural damage or neuronal loss, particularly in frontal lobe regions or corticobulbar pathways (Jose, Samuel, and Isabel 2020).

The assessment of PMR can provide valuable information for the prognosis and rehabilitation planning of TBI patients, as the presence and characteristics of the reflex may be indicative of the extent of neurological impairment and the potential for functional recovery (Sucksdorff et al. 2020).

### 4.7 Multiple Sclerosis

In patients with MS, PMR has been observed and may be related to the presence of demyelinating lesions or plaques affecting the neural pathways involved in the modulation of primitive reflexes (Gramespacher et al. 2020). PMR may be more prevalent in specific subtypes of MS, such as primary progressive MS or secondary progressive MS, where cortical and subcortical pathways are more extensively affected (Ragab et al. 2021).

The presence and characteristics of PMR in MS patients have been associated with disease progression, cognitive impairment, and the development of physical disabilities (Mantel et al. 2017). The assessment of PMR can provide valuable insights into the extent of neurological involvement and the potential impact on functional abilities in MS patients.

### 4.8 Other Neurological Conditions

PMR has been documented in various other neurological conditions, including brain tumors (Ho et al. 2013), epilepsy (Konicarova, Bob, and Raboch 2013), neurodevelopmental disorders (Bonti et al. 2024), metabolic and toxic encephalopathies (Xu et al. 2024). In these diverse neurological conditions, the presence and characteristics of PMR can provide valuable diagnostic and prognostic information, contributing to a better understanding of the underlying neurological impairments and potential treatment strategies (Zafeiriou, Tsikoulas, and Kremenopoulos 1995).

## 5 Clinical Implications and Future Directions

The assessment of PMR holds significant clinical utility and implications across various neurological disorders. This section discusses the potential applications of PMR in diagnosis, prognosis, and treatment monitoring, as well as the challenges and limitations that should be addressed in future research endeavors.

### 5.1 Diagnostic Applications

The presence of PMR in adulthood is generally considered a pathological sign, indicating the presence of underlying neurological dysfunction (di Biase et al. 2022). While PMR is not specific to any particular neurological disorder, its assessment can provide valuable diagnostic information when interpreted in the context of other clinical findings and neurological examinations.

In clinical practice, PMR can serve as a screening tool or an adjunctive diagnostic marker, prompting further investigations or neuroimaging studies to identify the underlying cause (Mattson 2023). For example, the presence of PMR in a patient with cognitive impairment or motor symptoms may support the diagnosis of a neurodegenerative disorder or cerebrovascular disease, and guide the selection of appropriate diagnostic tests (Harwood and Cowan 2020).

Additionally, the specific characteristics of PMR, such as its intensity, duration, and laterality, may provide insights into the localization or extent of the neurological impairment, aiding in the differential diagnosis and targeted evaluation (Faglioni 2020).

### 5.2 Prognostic Implications

Beyond its diagnostic value, PMR has shown promise as a potential prognostic marker in various neurological disorders. The presence, severity, and evolution of PMR over time can provide insights into disease progression, functional outcomes, and treatment response.

In neurodegenerative diseases, such as PD and AD, the presence and severity of PMR have been associated with cognitive decline, functional impairment, and the development of complications (Vallar 2020). Regular monitoring of PMR may help clinicians track disease progression and inform treatment decisions or adjustments (Harp et al. 2022).

In stroke and TBI, PMR can serve as a prognostic indicator of functional recovery and rehabilitation potential (Asadi et al. 2021). The persistence or resolution of PMR over time may reflect the extent of neuroplasticity and neural reorganization, guiding therapeutic interventions.

### 5.3 Treatment Monitoring and Response Evaluation

The assessment of PMR can also play a role in monitoring treatment response and evaluating the effectiveness of therapeutic interventions in various neurological disorders. By tracking changes in the presence, intensity, or characteristics of PMR, clinicians can gain insights into the impact of pharmacological or nonpharmacological treatments on the underlying neural pathways and inhibitory mechanisms.

In PD, for instance, the resolution or attenuation of PMR following dopaminergic treatment or deep brain stimulation may indicate a positive response and improved modulation of primitive reflexes (Drenth et al. 2020). Conversely, the persistence or exacerbation of PMR despite treatment may suggest the need for treatment adjustments or the exploration of alternative therapeutic approaches.

Similarly, in MS, PMR can be used as a marker to evaluate the efficacy of disease‐modifying therapies or rehabilitation interventions (Lochhead et al. 2020). Changes in PMR over time may reflect the extent of remyelination, neuroplasticity, or functional recovery, guiding treatment decisions and rehabilitation strategies.

### 5.4 Challenges and Limitations

PMR holds significant potential as a diagnostic and prognostic tool (Table 1). Despite the availability of various elicitation and grading methods, there is a lack of consensus on standardized protocols for assessing PMR (Alnajashi and Alyazidi 2023). Establishing universally accepted guidelines and protocols can improve the consistency and comparability of findings across different clinical settings and research studies. Efforts should be made to improve inter‐rater reliability through comprehensive training programs and the use of objective measurement techniques, such as electromyography or motion capture systems (Jankovic and Lang 2021). As PMR is a nonspecific sign, its diagnostic value may be limited in certain clinical scenarios (Ticku et al. 2022). Further research is needed to establish the specificity and sensitivity of PMR in various neurological disorders, as well as its ability to differentiate between different pathological conditions.

**TABLE 1:** Summary of PMR in neurological disorders.

Neurological disorder	Prevalence of PMR	Diagnostic ability	Prognostic ability	References
Parkinson's disease	Common clinical finding	Potential early detection marker; may precede cardinal motor symptoms	Associated with disease progression, cognitive impairment, and levodopa‐induced dyskinesias	Mandelbaum and Marks (2020), Sanchez‐Ramos, Ortoll, and Paulson (1996), Wibawa et al. (2023), and Cruccu et al. (2005)
Alzheimer's disease	More prevalent in later stages	Reflects cognitive and neurological changes; correlated with degree of cognitive impairment	Associated with functional decline	Burns, Jacoby, and Levy (1991), Sabayan et al. (2012), Karpenko and Keegan (2021), and Chiang et al. (2005)
Multiple system atrophy	Documented presence	Associated with degeneration of extrapyramidal and cerebellar systems	May aid in differentiating subtypes or phenotypes	Thomas (1994), Novak and Tabrizi (2011), Melillo et al. (2022), and Frank, Pari, and Rossiter (2006)
Stroke	Frequent observation, particularly with frontal lobe, basal ganglia, or corticobulbar pathway involvement	Associated with lesion location and extent; reflects neurological impairment severity	Provides insights into functional recovery and rehabilitation potential	Schott and Rossor (2016), Morningstar et al. (2005), Zhang et al. (2021), Damasceno et al. (2005), Rao, Jackson, and Howard (1999), and Dünser et al. (2018)
Vascular dementia	Observed presence	Related to subcortical lesions and white matter changes	May aid in differential diagnosis	Coebergh and Stanton (2020)
Traumatic brain injury	Common, especially with frontal lobe damage	Associated with injury severity and cognitive impairment	Indicator of chronic neurological deficits; useful for rehabilitation planning	Martello (2023), Miles et al. (2021), Wijdicks (2021), Jose, Samuel, and Isabel (2020), and Sucksdorff et al. (2020)
Multiple sclerosis	Observed presence, more prevalent in progressive subtypes	Related to demyelinating lesions affecting neural pathways	Associated with disease progression, cognitive impairment, and physical disabilities	Gramespacher et al. (2020), Ragab et al. (2021), and Mantel et al. (2017)

While PMR can provide valuable insights, it should be interpreted in conjunction with other clinical findings, neuroimaging data, and laboratory investigations (León‐Bravo, Cantarero‐Carmona, and Caballero‐Villarraso 2023). For example, when evaluating a patient with suspected PD, clinicians may consider the presence of other motor symptoms, such as tremors, rigidity, and bradykinesia, in addition to PMR. Similarly, in patients with suspected stroke, clinicians may consider the results of neuroimaging studies, such as CT or MRI scans, in conjunction with PMR. Developing integrative approaches that combine PMR with other diagnostic and prognostic markers can enhance the accuracy and reliability of clinical decision‐making. In addition, longitudinal studies involving repeated assessments of PMR in various neurological disorders are needed to fully elucidate its utility as a prognostic and monitoring tool (Lundblad et al. 2020).

By addressing these challenges and limitations through collaborative efforts among researchers, clinicians, and healthcare professionals, the clinical utility and reliability of PMR assessment can be enhanced, ultimately contributing to improved diagnostic accuracy, prognostic evaluation, and treatment monitoring in various neurological disorders.

## 6 Conclusion

PMR has emerged as a valuable clinical tool in the field of neurology, offering insights into the functional integrity of neural pathways and inhibitory mechanisms involved in the modulation of primitive reflexes in adulthood, to indicate an underlying neurological dysfunction. Through this comprehensive review, we have explored the clinical assessment techniques, neurophysiological basis, and diverse applications of PMR across a wide range of neurological disorders. From neurodegenerative diseases and cerebrovascular disorders to TBI and MS, PMR has demonstrated its potential as a diagnostic marker, prognostic indicator, and treatment monitoring tool.

By understanding the neural pathways, brain regions, and neurotransmitter systems involved in the modulation and suppression of PMR, researchers and clinicians can gain valuable insights into the specific neurological impairments associated with its reemergence in various conditions. In addition, the developmental aspects and inhibitory mechanisms underlying PMR provide a window into the neurodevelopmental and neurodegenerative processes that contribute to its manifestation.

As this primitive reflex holds the potential to become an increasingly valuable tool in the diagnostic armamentarium of clinicians. By leveraging the insights gained from PMR, we can potentially improve patient care, refine treatment strategies, and advance our understanding of the intricate workings of the human nervous system.

## Author Contributions

**Benxu Ma**: conceptualization, methodology, writing–original draft, writing–review and editing. **Jianying Zhang**: conceptualization, methodology, writing–original draft, writing–review and editing. **Yanlei Cui**: formal analysis, writing–original draft, writing–review and editing. **Huanmin Gao**: writing–original draft, writing–review and editing.

## Consent

The authors have nothing to report.

## Conflicts of Interest

The authors declare no conflicts of interest.

### Peer Review

The peer review history for this article is available at https://publons.com/publon/10.1002/brb3.70164.

## Data Availability

All data generated or analyzed during this study are included in this published article.
